# Supplementary material for: Applying Spinal Cord Organoids as a quantitative approach to study the mammalian Hedgehog pathway
Source: PLoS One. 2024 Jun 25;19(6):e0301670. doi: 10.1371/journal.pone.0301670 (PMC11198841; doi:10.1371/journal.pone.0301670)
Supplement: S1 File — (PDF) [file pone.0301670.s001.pdf]

Mar 15, 2024 Version 2

Derivation of Spinal Cord Organoids as a quantitative approach to study the mammalian Hedgehog Signalling V.2

DOI
dx.doi.org/10.17504/protocols.io.kxygx3r6og8j/v2

Markus Holzner[1], Anton Wutz[1], Giulio Di Minin[1]

[1]Institute of Molecular Health Sciences, Department of Biology, Swiss Federal Institute of Technology ETH Hönggerberg, Zurich, Switzerland

Giulio Di Minin: giulio.diminin@biol.ethz.ch

Giulio Di Minin
ETH Zurich

OPEN ACCESS

DOI: dx.doi.org/10.17504/protocols.io.kxygx3r6og8j/v2

Protocol Citation: Markus Holzner, Anton Wutz, Giulio Di Minin 2024. Derivation of Spinal Cord Organoids as a quantitative approach to study the mammalian Hedgehog Signalling. **protocols.io https://dx.doi.org/10.17504/protocols.io.kxygx3r6og8j/v2**Version created by **Giulio Di Minin**

License: This is an open access protocol distributed under the terms of the **Creative Commons Attribution License**, which permits unrestricted use, distribution, and reproduction in any medium, provided the original author and source are credited

Protocol status: Working
We use this protocol and it's working

Created: March 15, 2024

Last Modified: March 15, 2024

Protocol Integer ID: 96771

Keywords: Hedgehog, Neural tube, Organoids

Funders Acknowledgement:
Swiss National Science Foundation
Grant ID: 31003A_152814/1
Swiss National Science Foundation
Grant ID: 31003A_175643/1

Abstract

The Hedgehog (HH) pathway is crucial for embryonic development, and adult homeostasis. Its dysregulation is implicated in multiple diseases. Existing cellular models used to study HH signal regulation in mammals do not fully recapitulate the complexity of the pathway. Here we describe the procedure to derive Spinal Cord Organoids (SCOs) and we show how to apply them to quantitively study the activity of the HH pathway. During SCO formation, the specification of different categories of neural progenitors (NPC) depends on the intensity of the HH signal, mirroring the process that occurs during neural tube development. By assessing the number of NPCs within these distinct subgroups, we are able to categorize and quantify the activation level of the HH pathway. SCOs represent an accessible and reliable *in-vitro* tool to quantify HH signaling and investigate the contribution of genetic and chemical cues in the HH pathway regulation.

Materials

Differentiation Medium

	Volume
Advanced DMEM F12	25 mL
Neurobasal‰	25 mL
Knockout Serum Replacement (KSR)	5 mL
Pen Strep	500 µL
Glutamine	500 µL
2-mercaptoethanol	0.4 µL

Mix all media components and filter through a sterile filter with 0.22 µm pore size.
For day D3 add retinoic acid at a final concentration of 100 nM to the differentiation medium.

ESC base media

Component:	Volume
DMEM	Ad 500 mL
Fetal Bovine Serum	75 mL
MEM NEAA	5 mL
Sodium Pyruvate	5 mL
Pen Step	5 mL
2-mercaptoethanol	4 µL

All the components of the ESC base media are mixed and then filtered through a sterile filter unit with a pore size of 0.22 µm. The base media can be stored at 4 °C for up to 4 weeks.

ESC media with LIF and 2i

Component:	Volume
ESC base media	50 mL
LIF	100 µL (final units: 10’000)
PD0325901 (PD)	5 µL (final 1 µM)
CHIR99021 (CH)	15 µL (final 3 µM)

To prepare the final ESC maintenance media add PD, CH and LIF. The medium can be stored at 4 °C and used for up to 1 week.

Trypsin-EDTA solution

Thaw a 10X Trypsin bottle over night at 4 °C. Dilute the 100 mL Trypsin with 900 mL of sterile PBS and add 2 mL of 0,5 M EDTA solution (pH = 8, autoclaved, sterile). Mix well. Aliquots can be stored at -20 °C.

Gelatin

0,2 g of gelatin are dissolved in 500 mL of MilliQ Water and autoclaved to obtain a 0,2% solution. The solution can be stored at RT.

PFA solution

A 4% PFA solution is obtained by dissolving 20 g of PFA in 500 mL of PBS. Carefully heat the solution while stirring until PFA is dissolved. Aliquots can be stored at -20 °C.

Antibody Buffer (AB Buffer)

Component:	Amount
PBS	50 mL
BSA	0,5 g
Triton X	50 µL

Blocking Buffer

Component:	Amount
PBS	50 mL
BSA	0,5 g
Triton X	50 µL

Key resources

Reagents:

	Manufacturer	Catalogue Number
Advanced DMEM F12	Gibco	12634-010

Neurobasal‰ Medium	Gibco	21103-049
L-Glutamine 200 mM (100x)	Gibco	25030-024
Knockout Serum Replacement (KSR)	Gibco	10828010
2-mercaptoethanol	Sigma-Aldrich	M6250-250ML
BSA Fraction V (7,5 %)	Gibco	15260-037
Paraformaldehyde	Sigma-Aldrich	P6148-1KG
Dulbecco's Modified Eagle Medium	Gibco	41965-039
Fetal Bovine Serum	Biowest	S1810-500
MEM NEAA (100x)	Gibco	11140-035
Sodium Pyruvate (100mM)	Gibco	11360-070
LIF protein	Home made	
CHIR99021	AxonMedchem	252917-06-9
PD0325901	AxonMedchem	391210-10-9
DMSO	Genaxxon	M6323.0100
Retinoic Acid	Thermo Scientific	17110052
SHH protein	Home made	
Gelatine	Sigma	9000-70-8
Trypsin	Gibco	15090-046
EDTA	Sigma	E5134-250G
Bovine Serum Albumin	PanReac AppliChem	A1391,0100
Triton X	Sigma	T8787-250ML
Sucrose for microbiology	Sigma-Aldrich	84100-1KG
PBS pH 7.4 (1x)	Gibco	10010-015
Mounting media (Mowiol)	Home made	
DAPI	Thermo Scientific	D1306

Lab supplies

	Manufacturer	Catalogue Number
Sphericalplate 5D (Aggrewell)	Kugelmeiers	12038828
NunclonTM Delta Surface MW6	Thermo Scientific	140675

Stericup Millipore Express Plus	Sigma Aldrich	S2GPU05RE
Tissue Freezing Medium	Leica	14020108926
Tissue-Tek‰ Cryomold‰ (10 x 10 x 5)	Sakura	4565
SuperFrost‰ Plus	VMR	631-0108

Primary antibodies

Target	Host	Manufacturer	Catalogue Number	Dilution
Sox1	Goat	RNDsystems	AF3369	1:200
Olig2	Mouse	EDM Millipore	AB9610	1:200
Nkx2.2	Rabbit	DSHB	75.5A5	1:25
Pax6	Mouse	BioLegend	901301	1:200

Secondary antibodies

	Host	Manufacturer	Catalogue Number	Dilution
Alexa Fluor‰ 488-Anti rabbit	Donkey	Jackson ImmunoResearch	711-545-152	1:1000
Cy3-Anti mouse	Donkey	Jackson ImmunoResearch	115-165-003	1:1000
Cy5-Anti goat	Donkey	Jackson ImmunoResearch	705-175-147	1:1000

qPCR primers

qPCR Primer		Sequence 5’ to 3’
Pax7	FW<br>RV	GTGCCCTCAGTGAGTTCGATC<br>CACATCTGAGCCCTCATCC
Pax6	FW<br>RV	TAACGGAGAAGACTCGGATGA<br>AGCCGGGCGAACACATCTGGATAATGG
Olig2	FW<br>RV	GTACCTGGGGGCTTGACAAA<br>AACAAAGAGCTTCGCATCGC
Nkx2.2	FW<br>RV	TGCCCCTTAAGAGCCCTTTCT<br>CTCCTTGTCATTGTCCGGTG

Foxa2	FW<br>RV	GACTGGAGCAGCTACGCTCAT<br>TCCAGCGCCCACATAG
Sox1	FW<br>RV	TCTCCAACTCTCAGGGCTACA<br>CTTGACCAGAGATCCGAGGG
Gli1	FW<br>RV	GAATTCGTGTGCCATTGGGGG<br>GACTTCCGACAGCCTTCAA
Ptch1	FW<br>RV	TGACTGGGAAACTGGGAGGA<br>TGATGCCATCTGCGTCTACC
Sdha	FW<br>RV	TTCCGTGTGGGGAGTGTATTG<br>CAGGTCTGTGTTCCAAACCAT<br>TCC
Eif4a2	FW<br>RV	ACACCATCGGGGTCCATTCCC<br>CTGTCTTTTCAGTCGGGCG

Kits:

Kit name	Vendor	Catalogue Number
RNeasy‰ Mini Kit (50)	Qiagen	74104
QIAshredder (250)	Qiagen	79656
QuantiTect‰ Reverse Transcription Kit (200)	Qiagen	205313

mESC maintenance

1 **Note:** the procedure describes the necessary amounts for the expansion of mouse ESCs in a 3 cm dish. This dimension will provide enough cells to derive SCOs. Volumes can, however, easily be adapted according to the user's needs.

- Pre-coat the necessary number of wells of a 3 cm dish with 1 mL of 0,2% gelatin. Leave for at least 10 min at room temperature (RT).

Note: Alternatively, ESCs can be grown on feeder cells (e.g., mouse embryonic fibroblasts). To improve ESC attachment in the absence of feeder cells 0,5% of FBS can be added to the gelatin solution.

- split ES cells once colonies reach a confluency of 60-80%.

Note: Proper ESC growth conditions are crucial for the generation of SCOs. ESCs should be passed every 2 days. Inspect ESC culture and check for colony size and morphology. An overly dark center indicates a necrotic area within the colony due to cell overgrowth and must be avoided. Adjust plated cell numbers to match the specific needs of your ESC line. Loss of compactness and loose borders are signs of differentiation. In this case, a co-culture on feeder cells is suggested to enhance the pluripotency state of the ESCs.

- Aspirate the culture media, wash cells once with 2 mL of PBS.
- Incubate with an appropriate amount of Trypsin EDTA (200 µL per one well of a 3 cm dish) for 3-5 min at 37 °C.
- Once cells are detached from the plate, stop the reaction with 2 mL of ESC base media and transfer the cell suspension to a centrifuge tube.
- Spin the cells at 900 rpm for 5 min and resuspend the cells in ESC maintenance media.
- Count cells and plate 400k ESCs on a gelatin pre-coated culture dish.

Note: For neuronal EB induction, resuspend the ES cells after centrifugation in the differentiation medium to avoid a carry-over of 2i and LIF.

Derivation of Spinal Cord Organoids (SCOs)

2 **Day 1. ESC plating in AggreWell plate for nEB formation**

Timing: 1 to 2 hours, depending on the number of samples.

Per each sample, prepare two wells of an AggreWell Plate.

- Incline the AggreWell plate to an angle of 30-40° and slowly add 500 µL of differentiation medium to the bottom of each well. Make sure to evenly distribute the media and avoid bubble formation. Bubbles can be removed by pipetting or lightly shaking.
- Plate 50k to 150k ESCs per well. Resuspend cells in 1 mL of differentiation media per sample, mix, and transfer cells into the AggreWell plate.

Note: The final dimension of nEBs is crucial for neuralization and pattering of later SCOs. The amount of ESCs plated in the AggreWell wells must therefore be adjusted and optimized according to the used ESCs.

- Distribute the cells by pipetting up and down slowly and avoid bubble formation.
- Seal the plate with Parafilm and spin for 5 min at 900 rpm. This step is optional, will however increase the size homogeneity of later formed nEBs.
- Incubate plates at 37 °C and 5 % CO_2 until Day 3.

3 **Day 3. Transfer of nEBs to a 10 cm culture dish**

Timing: 15 min per sample

- Transfer each sample (2 wells of the AggreWell plate) into one 10 cm dish. Use low-attachment plates to prevent nEBs from attaching to the plate.
- Add 10 mL of differentiation media, supplemented with 100-200 nM retinoic acid to a 10 cm plate.

Note: RA activity is strongly different between formulations and according to the storing conditions. We try to avoid freeze-thaw cycles. The optimal RA concentration should be defined by users.

- With a cut tip and a P1000 pipet wash the nEBs out of their wells by slowly pipetting up and down, approximately 5 times.

Note: When transferring the nEBs always use P1000 tips with the front cut off. The increased opening will help to maintain the nEBs structural integrity while handling.

- Transfer the nEBs to their respective 10 cm plate.
- Evenly distribute nEBs in the plate by gently rocking the plates in circles, drawing an 8 when looked at from above.
- Carefully transfer the plates back to the incubator.
- Incubate plates at 37 °C and 5 % CO_2.

Note: From this point on we call the nEBs now Spinal Cord Organoids (SCOs)

4 **Day 4. Patterning of the dorsal to ventral axis.**

Timing: 10 min

ES cells have now reached the stage of the neuronal plate. To trigger the dorsal to ventral axis patterning, samples can be treated with the SHH protein or hedgehog agonists like SAG or Purmorphamine (PMP).

- To induce specification, add SHH at 200 ng/mL or PMP at a final concentration of 1 to 2 µM. The negative control is maintained in only retinoic acid.
- Transfer plates back into the incubator and ensure an even distribution of SCOs among the whole plate.
- Incubate plates at 37 °C and 5 % CO_2.

5 **Day 6. End of the Experiment and sample preparation for either qPCR analysis or IF staining.**

SCOs are collected for downstream analysis

Note: Ventral markers are already detected after 24 h of SHH treatment and remain expressed up until 48 h of SHH treatment. Thereafter (day 8), NPCs start to differentiate and will express neuronal markers.

Preparing SCOs for transcriptional analysis

6 **Lysis of the samples**

- Prepare one microcentrifuge tube (2 mL) for each sample. Collect SCOs in the center of the 10 cm plate by slowly swirling the plate in small circles.
- Transfer the SCOs to the Eppendorf tube with a P1000 pipet (and a cut tip) and let them sediment by gravity.
- Remove supernatant and wash SCOs twice with each 2 mL of PBS, letting the SCOs sediment by gravity in between each washing step. Remove the supernatant.

7 **RNA extraction**

- Resuspend the SCOs in the lysis buffer of your preferred RNA extraction method. We use the Quiagen RNeasy kit with the following adjustments.
- Add 500 µL of lysis buffer (RLT) per sample and dissociate SCOs by pipetting.
- Transfer samples to a spin column of the QIAshredder and collect the lysates by a short centrifugation (15 s, max speed).
- Follow the RNA extraction protocol as suggested by the manufacturer and include the optional on-column DNAse digestion.
- Extracted RNA is eluted with 40 µL of water.

8 **cDNA preparation**

- cDNA is prepared with the Qiagen QuantiTect Reverse Transcription Kit following the manufacturer's instructions.

Immunofluorescence analysis of SCO sections

9 **Sample preparation for immunofluorescence analysis**

Timing: 4 h

Note: To maintain the SCOs structural integrity, avoid unnecessary pipetting as much as possible.

- Transfer SCOs into microcentrifuge tubes by first collecting them in the center of the culture dish swirling the plate in small circles, and then taking them up with a cut, BSA-coated P1000 tip.

Note: Coat microcentrifuge tubes (2 mL Eppendorf tubes) with a BSA solution; discard BSA solution. Do the same for tips that are used to transfer the SCOs. This will prevent SCOs from sticking to the plastic of the tubes and tips.

- Wash SCOs twice with ice-cold PBS. Let SCOs sink to the bottom of the microcentrifuge tube by gravity alone, remove the supernatant and add 1 mL of ice-cold PBS to each sample. Repeat this process once more.

- Fixing SCOs. Remove PBS and add 1 mL of a freshly prepared 4 % PFA solution to each sample. Incubate for 30 min at 4 °C, shaking at about 100 rpm.
- Wash the fixed SCOs twice with each 1 mL of ice-cold PBS just as done before. After the second wash, remove almost all PBS but leave ca. 150 µL in each tube.
- Cryoprotect the SCOs in sucrose. For best results, the sucrose concentration is increased stepwise from 10 to 30%. Start by adding 1 mL of a 10% sucrose solution in PBS to each sample. You will notice, that the SCOs will now float at the interface between the sucrose solution and PBS. Incubate the samples again at 4 °C, shaking until the SCOs are saturated with sucrose and have sunk to the bottom of the microcentrifuge tube. This will take about 30 min. Repeat with 20% and 30% of sucrose solutions always leaving ca. 150 µL on top of the SCOs after each step.
- Embedding and freezing. The tissue freezing medium is very viscous and is pipetted best with a cut P1000 tip. Add about 1 mL of the freezing medium to each sample. Swirl and resuspend SCOs stirring, not pipetting with a P200 tip inside the tube. Then transfer the SCOs into a Cryomold®. Avoid air bubbles and evenly distribute SCOs over the whole area of the Cryomold®. Top off with additional tissue freezing medium until the mold is filled. Transfer the container onto a cold metal block to freeze and then store samples at -80 °C.

Note: Freezing SCOs for later cryosections is best performed directionally, bottom to top. Therefore, add dry ice to a box and place a metal block inside. The block will cool down and serve as platform where the Cryomolds® can be placed for freezing.

10 **Preparing cryosections**

Timing 1h per sample

- Cryosections are prepared with a cryotome. Following the device specific instructions, cut 10-15 µm thick slices and collect them on SuperFrost® Plus slides.
- Let the sections dry at room temperature for 1 h. Cryosections can be stored at –80 °C.

11 **Immunofluorescence staining**

Timing 2 Days

- Let the slides warm up to RT.
- Rehydrate cryosections for 30 min in PBS.
- Permeabilization/Blocking. Incubate samples in PBS with 0,3% Triton X and 10% Donkey Serum for 30 min at RT.
- Primary Antibody (AB) incubation. Prepare a master mix of 300 µL per slide. Primary antibodies are diluted in AB buffer and mixed well. The AB solution is added to each slide, and a piece of parafilm (equal size as the slide) is added carefully on top. The slides are incubated overnight at 4 °C in a humidified chamber.
- Samples are washed three times with PBS Triton X (0,1%) for 5 min each.
- Secondary AB incubation. Secondary ABs and Dapi are diluted in AB buffer. Again 300 µL of AB solution is added to each slide, protected with a piece of parafilm, and incubated at RT, in the dark, for 30 min.
- Samples are washed twice with PBS Triton X (0,1%) for each 5 min and once with PBS for 5 min.

- Mount samples with Mowiol and leave to dry at RT, in the dark, overnight. Samples can now be analyzed with a fluorescence microscope.

Note: We generally acquire SCOs picture using a 20X objective. For each condition, at least 10 independent SCOs are acquired and counted.
