# Supplementary material for: Applying Spinal Cord Organoids as a quantitative approach to study the mammalian Hedgehog pathway
Source: PLoS One. 2024 Jun 25;19(6):e0301670. doi: 10.1371/journal.pone.0301670 (PMC11198841; doi:10.1371/journal.pone.0301670)
Supplement: Attachment — Submitted filename: Response to the Reviewers.docx [file pone.0301670.s003.docx]

**Response to the editorial comments**

Thank you for submitting your manuscript to PLOS ONE. After careful consideration, we feel that it has merit but does not fully meet PLOS ONE’s publication criteria as it currently stands. Therefore, we invite you to submit a revised version of the manuscript that addresses the points raised during the review process.

Again, we would like to thank the reviewers and the editor for their comments, which have helped us strengthen our manuscript further. We responded to all the individual points raised by Reviewer #1. As suggested, we modified the images shown in Fig1D to better show SMO translocation at the PC upon SHH treatment.

**Response to Reviewer #1**

The authors have addressed all concerns previously raised and significantly improved the quality of the study.

Response: We thank the reviewer for his positive assessment of our revision.

I just have 2 minor points that should be easily tackled without the need of further experiments. Please, see below.

Unless I'm reading this wrong, the claim in lines 210-211 ("Additionally, NKX2.2 represses the Olig2 gene, which iscoherent with the in vivo situation") is not supported by the results. Actually Fig 2E-F shows a continuous increase OLIG2 AND NKX2.2 expression (if at the highest [SHH] NKX2.2 was highest and OLIG2 was lower than a lower [SHH] one could make that extrapolation (not a claim and just based on in vivo knowledge), but actually OLIG2 never decreases in that plot. So I would just indicate that the expression of both ventral progenitor markers increases according to increasing [SHH], without entering in OLIG2 inhibition by NKX2.2 (maybe even higher [SHH] would have needed to be tested, or the expression of the markers checked a few days later).

Response: We have followed the reviewer’s comment and modified the phrasing as suggested (line 210).

Also, it's a pity that the images in Fig 1D poorly show the primary cilia, especially upon SHH treatment. Ideally, better images should be shown, if possible.

Response: We provide new images (Fig1D) that better support SMO translocation upon SHH treatment. Compared to experiments performed in fibroblast cells, SCO sections are not optimal for characterizing SMO translocation at the primary cilium due to two drawbacks of the methodology: 1) the majority of NPCs in SCOs are in a proliferative stage, which means that either they do not have any PCs or the PCs are not long enough to be distinguished clearly (as they are more associated with the G0 phase). 2) the sections that are produced during the slicing of the EBs are usually around 10-15μm thick, which may not include the necessary planes to detect the PCs entirely.

**Response to Reviewer #2**

The authors have adequately addressed the requested points. The manuscript has been modified to better describe the cell lines used and the background material, as well as better highlight the use of AggreWell in their protocol compared to the existing literature.

Response: We thank the reviewer for the specific comments and the positive evaluation of our study.
